# The remanence ratio in CoFe_2_O_4_ nanoparticles with approximate single-domain sizes

**DOI:** 10.1186/s11671-016-1691-3

**Published:** 2016-10-22

**Authors:** Shitao Xu, Yongqing Ma, Bingqian Geng, Xiao Sun, Min Wang

**Affiliations:** 1 Anhui Key Laboratory of Information Materials and Devices, School of Physics and Materials Science, Anhui University, Hefei, 230601 People’s Republic of China; 2 School of Physics and Electronic Information, Huaibei Normal University, Huaibei, 235000 People’s Republic of China

**Keywords:** CoFe_2_O_4_ nanoparticles, Remanence ratio, Dipolar interaction, Surface spins

## Abstract

Approximately single-domain-sized 9-, 13-, and 16-nm CoFe_2_O_4_ nanoparticles are synthesized using the thermal decomposition of a metal-organic salt. By means of dilution and reduction, the concentration, moment, and anisotropy of nanoparticles are changed and their influence on the magnetic properties is investigated. The relation of *M*_r_/*M*_s_ ∝ 1/lg*H*_dip_ is observed, where *M*_r_/*M*_s_ is the remanence ratio and *H*_dip_ is the maximum dipolar field. Especially, such relation is more accurate for the nanoparticle systems with higher concentration and higher moment, i.e., larger *H*_dip_. The deviation from *M*_r_/*M*_s_ ∝ 1/lg*H*_dip_ appearing at low temperatures can be attributed to the effects of surface spins for the single-phase CoFe_2_O_4_ nanoparticles and to the pinning effect of CoFe_2_O_4_ on CoFe_2_ for the slightly reduced nanoparticles.

**Graphical Abstract:**
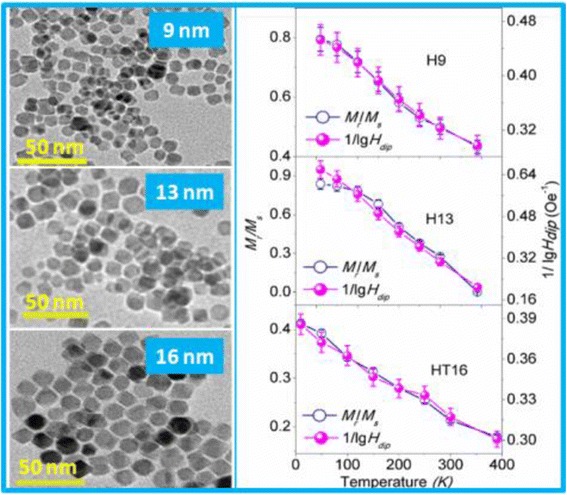
Approximately single-domain-sized 9-, 13-, and 16-nm CoFe_2_O_4_ nanoparticles were synthesized and then the concentration, moment, and anisotropy of these NPs were changed. The correlation of *M*_r_/*M*_s_ ∝ 1/lg*H*_dip_ was observed, independent of the size, concentration, moment, and anisotropy, and especially, such correlation is more accurate for the nanoparticle systems with higher concentration or moment, i.e., stronger dipolar interaction, which has not been reported before as far as we know.

Approximately single-domain-sized 9-, 13-, and 16-nm CoFe_2_O_4_ nanoparticles were synthesized and then the concentration, moment, and anisotropy of these NPs were changed. The correlation of *M*_r_/*M*_s_ ∝ 1/lg*H*_dip_ was observed, independent of the size, concentration, moment, and anisotropy, and especially, such correlation is more accurate for the nanoparticle systems with higher concentration or moment, i.e., stronger dipolar interaction, which has not been reported before as far as we know.

## Background

Nanoscale magnetic materials often exhibit novel properties, differing from those of their bulk polycrystalline counterparts [1–5], as a result of several effects including the finite size effect, surface effect, and interparticle interaction [6–8]. These effects affect the magnetic properties and the magnetic ordering state of nanoparticles (NPs) individually, and sometimes synergetically which usually occurs in the dense magnetic NPs. One typical phenomenon of the size effect is that the coercivity (*H*_c_) reaches the maximum as the particle size (*D*) decreases to a single-domain critical dimension *D*_c_, and then reduces monotonically to zero when *D* is further decreased to a certain size below *D*_c_ [9]. Concomitantly, the NPs exhibit the superparamagnetic behavior with the theoretical remanence (*M*_r_) to saturation (*M*_s_) magnetization ratio (*M*_r_/*M*_s_) being zero. Surface spin is another factor to affect the magnetic properties for the nano-sized magnetic materials. The total magnetization of a nanoparticle composes of the surface and core spins [10, 11], which is known as the core-shell magnetization model. Surface spins reduce the magnetization of a magnetic nanoparticle due to the disorder of spins at the nanoparticle surface, and the disordered surface spins lower the critical magnetic ordering temperature of magnetic nanoparticles compared with that of the bulk material [12]. Below a certain temperature, the canted surface spins freeze into a spin-glass-like state and the hysteresis loops obtained after a field cooling (FC) shift, as a consequence of the unidirectional anisotropy resulting from the coupling between the disordered surface layer and core spins [6, 13, 14]. Furthermore, the interaction between the surface spins of different particles enhances the effective anisotropy [7, 15], making the surface anisotropy constant many orders higher than that of the bulk material [16].

Apart from surface spins, the interparticle dipolar interaction (IPDI) widely exists in the magnetic NPs; it plays a complex role in magnetic properties. For example, it has been suggested that the IPDI enhances *H*_c_ because of additional induced anisotropy [17], while the opposite conclusion has also been observed [18]. A strong IPDI decreases the *M*_r_/*M*_s_ ratio, which has been proved both theoretically and experimentally [18–23]. Furthermore, it is well known that the strength of IPDI depends on the concentration of magnetic NPs and obviously affects the magnetic ordering states: in a heavily diluted system with low concentration of magnetic NPs, the system exhibits superparamagnetism [24], while in a dense system with high concentration, the NPs exhibit the super-spin glass (SSG) states at low temperature [25, 26].

So far, the effects of IPDI on the magnetic properties have not been comprehensively investigated, instead they have been reported piecemeal by different researchers. The strength of IPDI, which can be expressed by the maximum dipolar field *H*_dip_, defined as *H*_dip_ = 2 *μ*/*d* ^3^, where *μ* is the particle moment (*μ* = *M*_s_ × *V*_m_; *M*_s_ is saturation magnetization and *V*_m_ is magnetic grain volume) and *d* is the distance between particles (center to center), which is inversely proportional to the NP concentration. The purpose of the present work is to systematically reveal the effects of *H*_dip_ on the *M*_r_/*M*_s_ ratio for the 9-, 13-, and 16-nm magnetic NPs with different concentrations, particle moments, and anisotropies.

## Methods

### Experimental Procedure

#### Preparation of CoFe_2_O_4_ NPs

Co(acac)_2_ (97 %; acac is acetylacetonate), Fe(acac)_3_ (98 %), benzyl ether (97 %), oleic acid (90 %), and oleylamine (80–90 %) were mixed in a 1000-ml three-necked round-bottom flask by magnetic stirring under a flow of nitrogen (99.999 %). The mixture was heated at 120 °C for 0.5 h to remove air and moisture, at 200 °C under reflux for 2 h, and then at 290 °C for 1 h. After the mixture was cooled naturally to room temperature, absolute ethanol was added to produce a precipitate. The precipitate was separated via centrifugation and then washed with absolute ethanol several times to obtain CoFe_2_O_4_ NPs. By varying the intermediately treating temperature, we prepared 9-, 13-, and 16-nm CoFe_2_O_4_ NPs.

#### Dilution of CoFe_2_O_4_ NPs in a SiO_2_ Matrix

In order to change the interparticle distance, some of the 9- and 13-nm CoFe_2_O_4_ NPs were diluted in a SiO_2_ matrix with different concentrations, because the interparticle distance is inversely proportional to the concentration. CoFe_2_O_4_ NPs were added to a solution of cyclohexane (400 ml), polyethylene glycol (25 ml), tetraethyl orthosilicate, and ammonia, and then stirred mechanically for 24 h. Ethanol was added to form a precipitate. The precipitate was isolated by centrifugation and then washed with ethanol and water to remove unreacted molecules. The precipitate was dried at 80 °C for 6 h to obtain the diluted CoFe_2_O_4_ NPs by SiO_2_. By increasing the relative content of CoFe_2_O_4_, we prepared the CoFe_2_O_4_ NPs with low, moderate, and high concentrations, and the samples are referred to as L9, M9, and H9 for 9-nm NPs and as L13, M13, and H13 for 13-nm NPs.

#### Reduction of CoFe_2_O_4_ NPs

In order to change the moment and anisotropy of NPs, the reduction reactions were performed in the H_2_/N_2_ atmosphere (500 sccm, 96 % N_2_ + 4 % H_2_) to prepare a composite of CoFe_2_O_4_ and CoFe_2_ alloy, because the CoFe_2_ alloy is a typical soft ferromagnet with high moment and small anisotropy, compared with CoFe_2_O_4_. To avoid the aggregation of NPs during reduction, the 16-nm NPs were first diluted or separated by SiO_2_ and then reduced at low, moderate, and high temperatures at 300, 400, and 500 °C for 4 h, and the obtained samples are denoted as LT16, MT16, and HT16.

#### Characterization

The crystal structure of the products was determined by X-ray diffraction (XRD) using an X-ray diffractometer (DX-2000 SSC) with Cu Kα irradiation (*λ* = 1.5406 Å) in the scanning range 20°–80° with a step size of 0.02°. (High-resolution) transmission electron microscopy ((HR)TEM; JEOL, JEM-2100) was used to observe the morphology characteristics. Magnetic measurements were carried out using a superconducting quantum interference device PPMS system (Quantum Design, PPMS EC-II).

## Results and Discussion

### Crystal Structure and Morphology

**Fig. 1:**
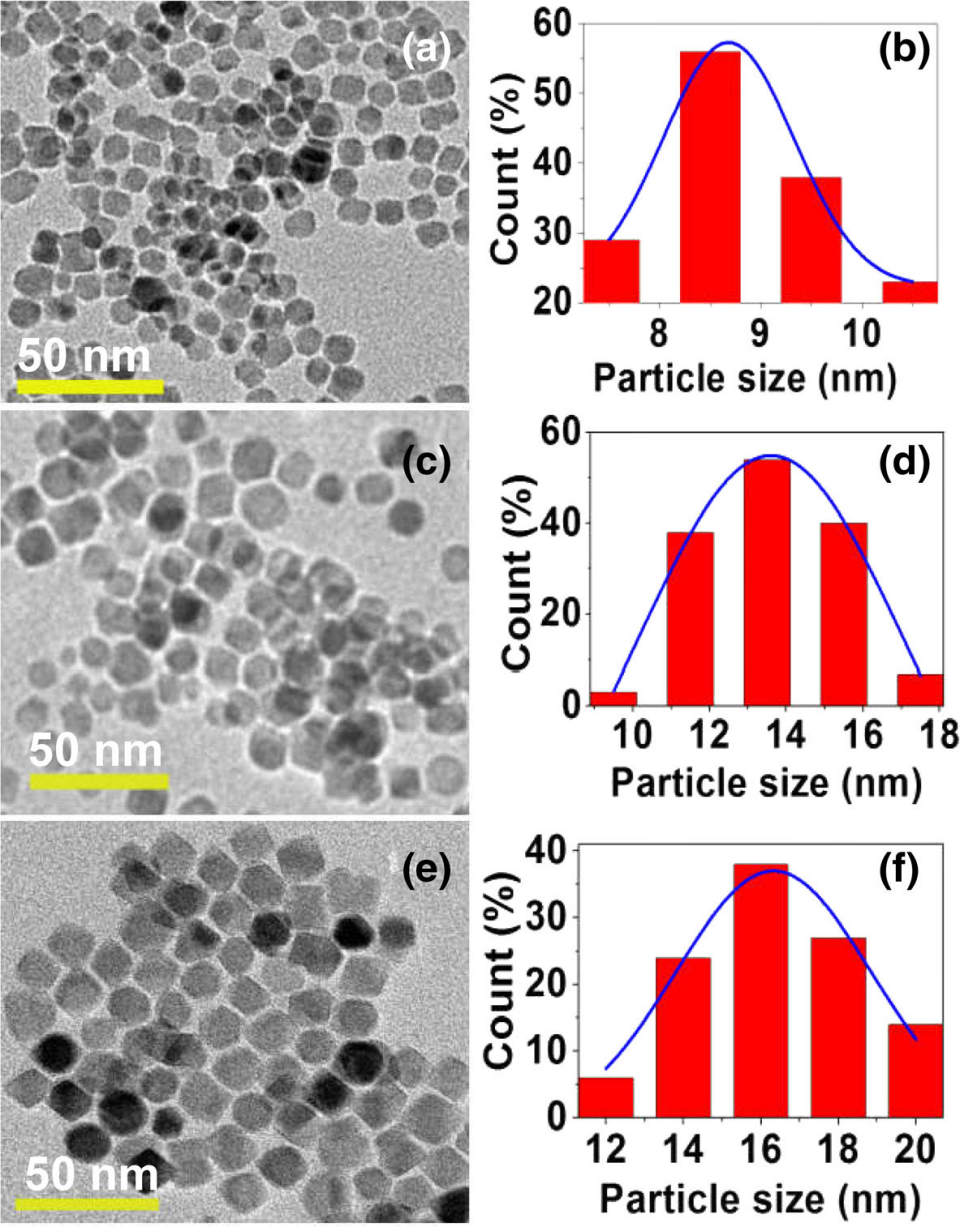
TEM images (*left*) and size distribution histograms with Gaussian-fitteng curve (*solid line*) (*right*) for 9-nm (**a** and **b**), 13-nm (**c** and **d**) and 16-nm (**e** and **f**) CoFe_2_O_4_ NPs

TEM images and size histograms in Fig. 1 show that the NPs are ca. 9, 13, and 16 nm. Most of 9- and 13-nm NPs exhibit the spherical-like morphology, while some 16-nm NPs exhibit rhombohedral and quadrate shapes. The size distribution range is ca. 2 nm for the 9-nm NPs and 4 nm for 13- and 16-nm NPs.

**Fig. 2:**
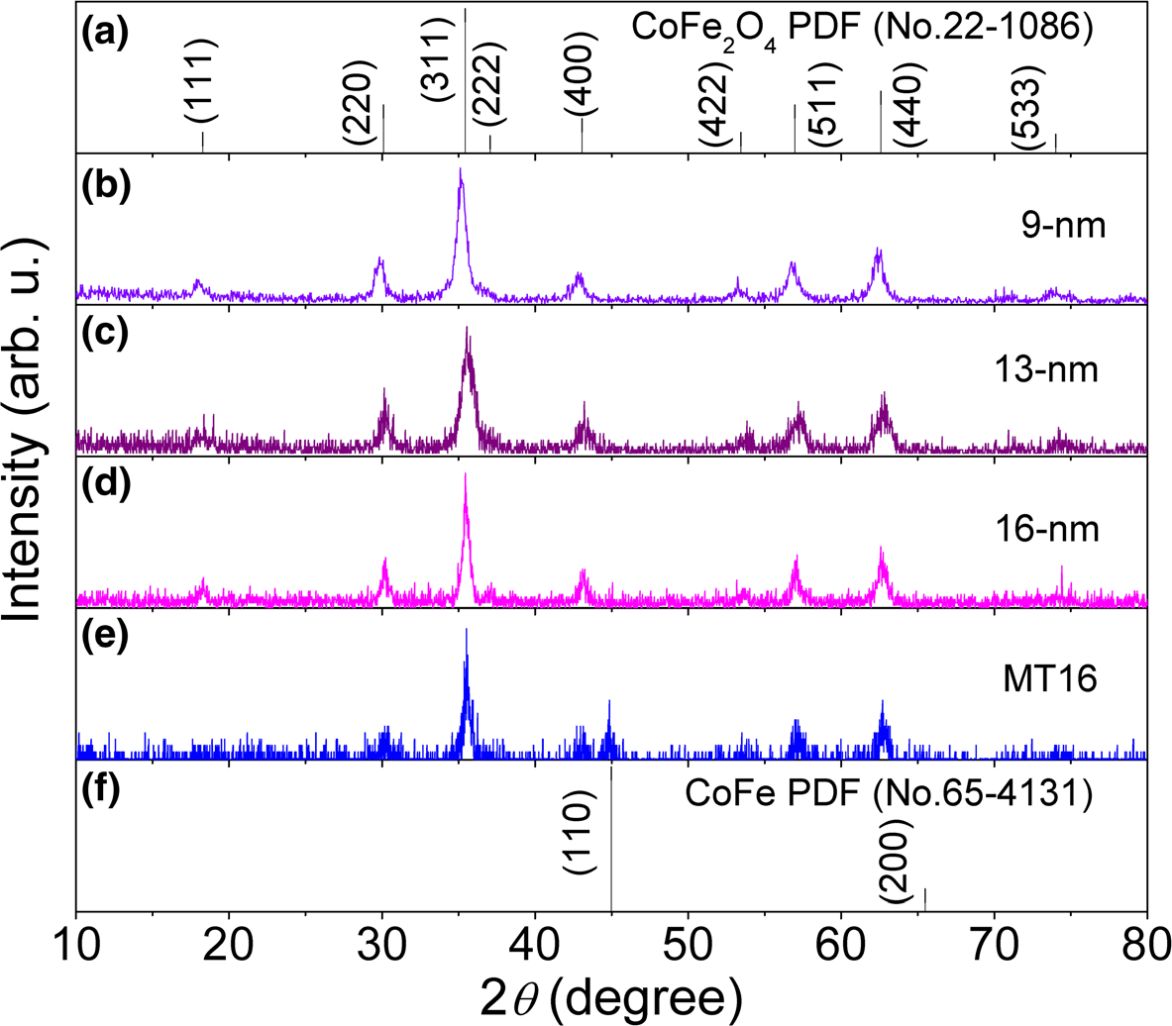
XRD patterns for as-prepared 9-nm (**b**), 13-nm (**c**), and 16-nm (**d**) CoFe_2_O_4_ NPs, and the MT16 sample (**e**) as well as the standard PDF cards of CoFe_2_O_4_ (No. 22-1086) (**a**) and CoFe_2_ (No. 65-4131) (**f**)

The XRD experiments have been performed on all samples, and herein, we show the results of several samples representatively. As shown in Fig. 2b–d, the as-prepared and undiluted 9-nm (the H9 sample), 13-nm (the H13 sample), and 16-nm CoFe_2_O_4_ NPs are single phases and have the cubic spinel structure, according to the standard powder diffraction file (PDF) of CoFe_2_O_4_ (No. 22-1086) in Fig. 2a. For the diluted 16-nm NPs after reduction at 400 °C, i.e., the MT16 sample, the extra diffraction peaks, besides those from CoFe_2_O_4_, can be assigned to the reflection from (110) and (200) crystallographic planes of the CoFe_2_ alloy, according to the PDF card of CoFe_2_ (No. 65-4131) in Fig. 2f. This XRD result indicates that CoFe_2_O_4_ is partially reduced to CoFe_2_ due to the reaction: CoFe_2_O_4_ + 4H_2_ → CoFe_2_ + 4H_2_O [27]. To observe the existence of CoFe_2_ in the reduced sample, the (HR)TEM was ever performed on the undiluted sample. The lattice fringes of CoFe_2_ can be observed at the surface of particle, i.e., CoFe_2_ exists at the outer layer while CoFe_2_O_4_ exists in the inner of particle.

### Magnetic Properties

**Fig. 3:**
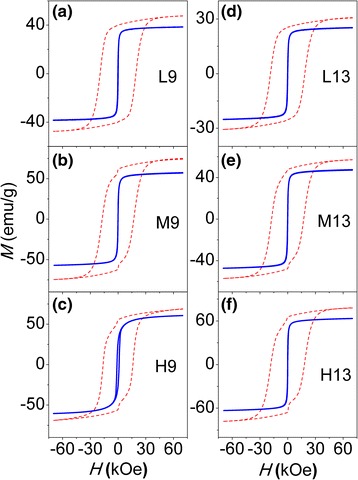
*M*(*H*) loops at 10 K (*dashed lines*) and 390 K (*solid lines*) of 9-nm NPs: L9 (**a**), M9 (**b**), and H9 (**c**); 13-nm NPs: L13 (**d**), M13 (**e**), and H13 (**f**)

**Fig. 4:**
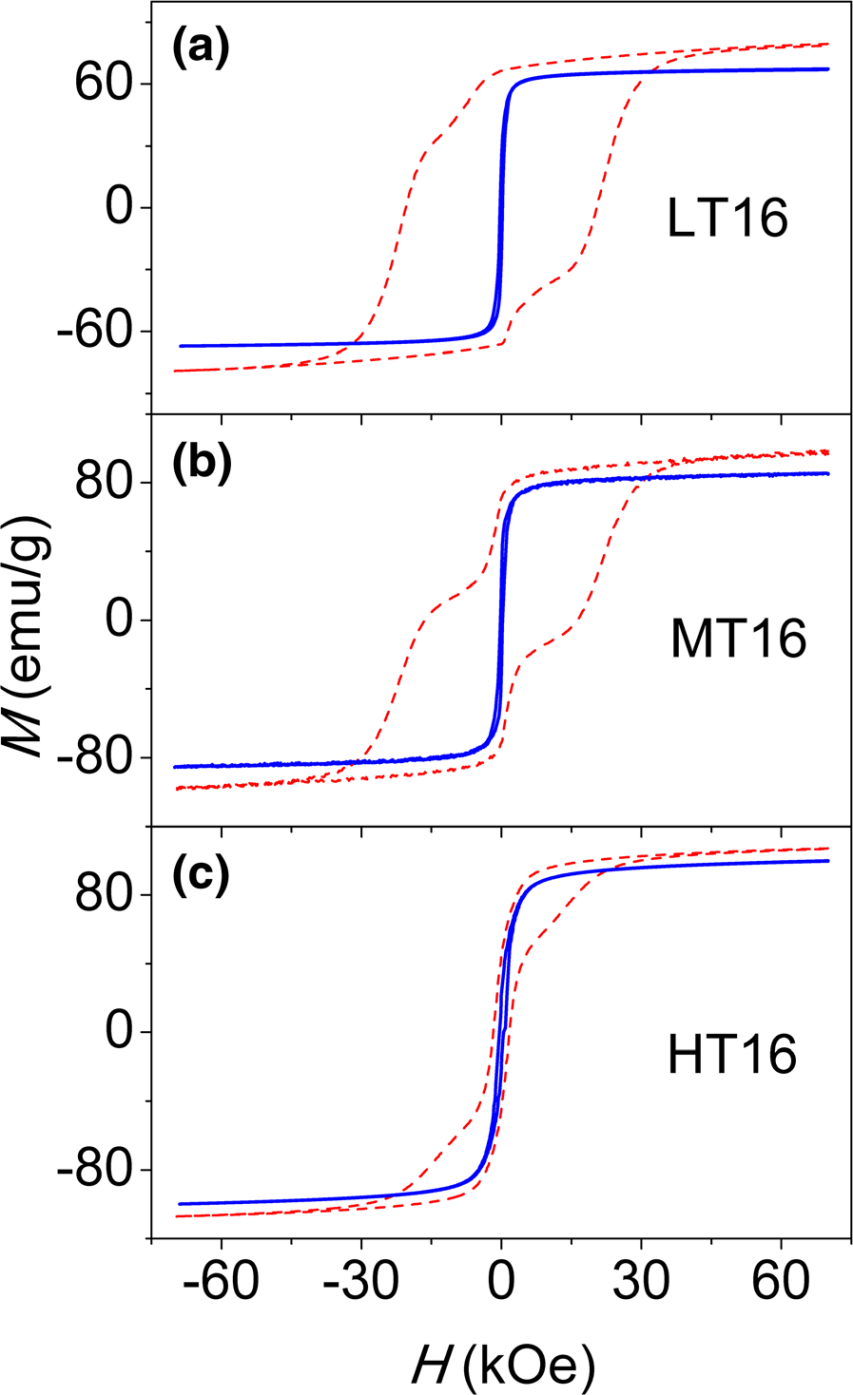
*M*(*H*) loops of diluted 16-nm NPs with low, moderate, and high reducing temperatures, i.e., LT16 (**a**), MT16 (**b**), and HT16 (**c**)

The dependence of the magnetization (*M*) of all samples on the applied magnetic field (*H*), i.e., *M*(*H*) loop (−70 kOe < *H* < 70 kOe) was measured at temperatures of 10, 50, 100, 150, 200, 250, 300, and 390 K. Representatively, the *M*(*H*) loops recorded at 10 and 390 K were shown in Fig. 3 for 9- and 13-nm NPs with different concentrations and in Fig. 4 for diluted 16-nm NPs with different reducing temperatures. From these loops, the coercivity (*H*_c_), saturation magnetization (*M*_s_), and remanence (*M*_r_) to saturation magnetization ratio (*M*_r_/*M*_s_) values at different temperatures can be obtained. In the case of the CoFe_2_O_4_ NPs with moderate (Fig. 3b, e) and high (Fig. 3c, f) concentrations, the loops recorded at 10 K show a jump around *H* = 0; this phenomenon is common for magnetic NPs and can be assigned to the reorientation of surface spins around NPs [28, 29]. However, in the case of the CoFe_2_O_4_ NPs with low concentration, as shown in Fig. 3a, d, the 10-K loops become smooth which is characteristic of a single-phase hard magnet, and the possible reason is that the surface spins are strongly pinned by the SiO_2_ matrix.

For the diluted and reduced 16-nm NPs, containing CoFe_2_O_4_ and CoFe_2_ phases, as shown in Fig. 4, the jump around *H* = 0 becomes higher as the reducing temperature increases, as a result of the increase in the relative CoFe_2_ content [30, 31]. Herein, the jump is attributable to the different reversal fields of hard CoFe_2_O_4_ and soft CoFe_2_, which is an indicative of no exchange-coupling occurring between soft and hard species, because in an exchange-coupled system, the magnetization could show an equivalent reversal behavior over the whole temperature region and the loop should be as smooth as that of the single-phase hard magnet. With increasing temperature, the anisotropy field of CoFe_2_O_4_ decreases markedly, so the average reversal fields of hard and soft phases may be similar, resulting in the single-phase behavior of the loops (not shown here).

**Fig. 5:**
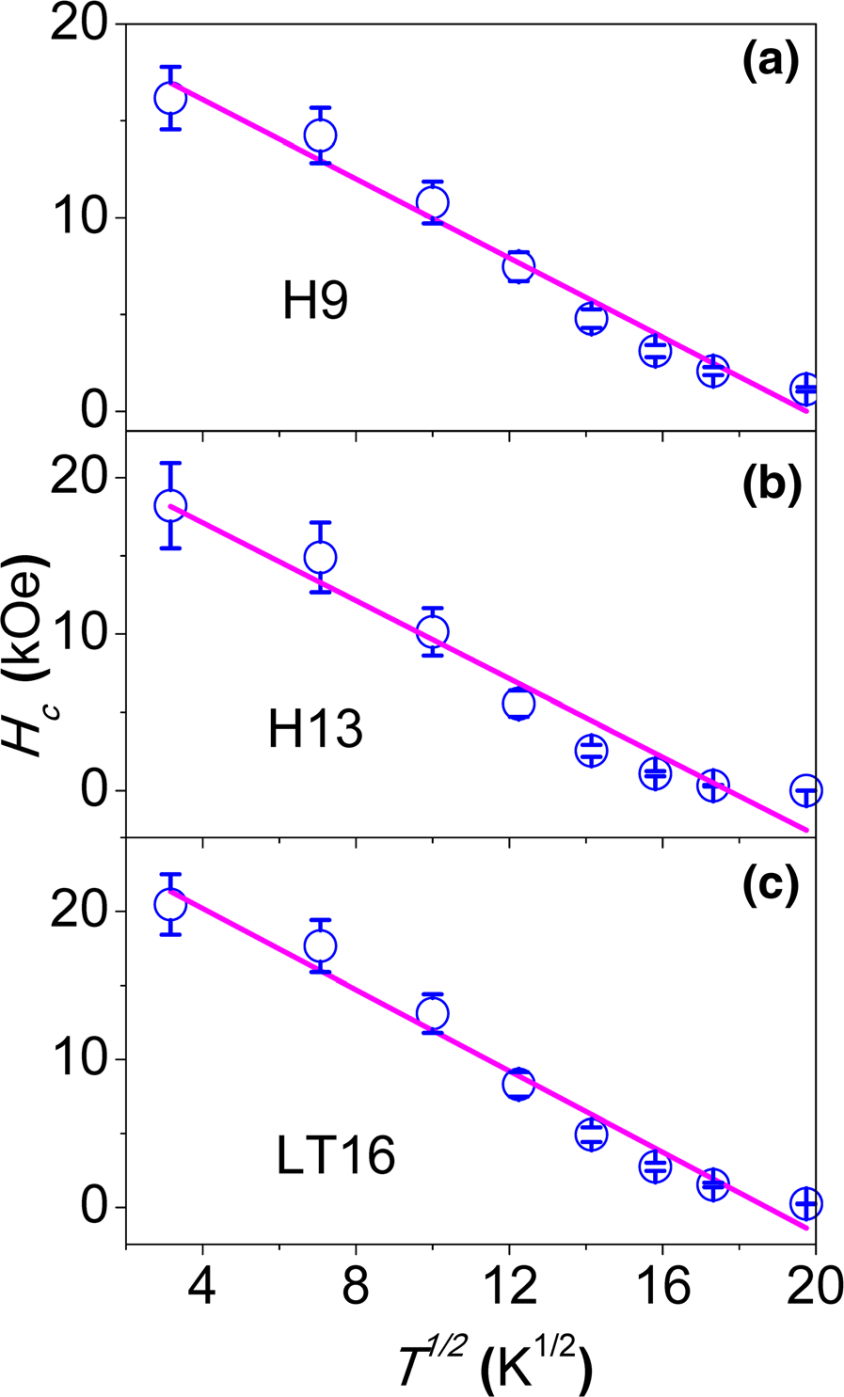
Coercivity *H*_c_ versus square root temperature for samples (*circles*) H9 (**a**), H13 (**b**), and LT16 (**c**) where the *solid line* is the fit to the experimental data points according to Kneller’s law

The undiluted 9- and 13-nm NPs, i.e., H9 and H13, as well as the 16-nm NPs reduced at 300 °C (LT16) have the *H*_c_ values of 20.0, 18.8, and 20.5 kOe at 10 K, smaller than 23.8 kOe (10 K) for our previously reported 10.7-nm CoFe_2_O_4_ NPs [32] which particle size is close to the critical size of a single domain [33]. These *H*_c_ values are much larger than that for the bulk CoFe_2_O_4_ (6.8 kOe at 10 K) [34], because on the one hand, the particles are approximately single-domain sized, and on the other hand, the interaction between surface spins induces extra anisotropy and hence enhances *H*_c_, which will be further discussed below. As the temperature increases, *H*_c_ monotonically decreases. The decrease in coercivity can be attributable to thermal fluctuations of the blocked moment, across the anisotropy barrier. For an assembly of non-interacting single-domain magnetic nanoparticles with uniaxial anisotropy, the coercivity can be written in the form of simple model of thermal activation of particle moments over the anisotropy barriers (Kneller’s law) as [6, 35] *H*_c_ = *H*_c0_[1 − (*T*/*T*_B_)^1/2^], where *H*_c0_ is the value of *H*_c_ at 0 K and *T*_B_ denotes the blocking temperature. The *H*_c_ values of all samples can be fitted to Kneller’s law in the temperature range of 10–390 K. Representatively, the experimental (solid circles) and fitting curves (solid lines) of the samples H9 (a), H13(b), and LT300 (c) are plotted in Fig. 5. The obtained fitting parameter *T*_B_ will be used to calculate the volume of magnetic grain, *V*_m_, as discussed below.

In the case of non-interacting and randomly oriented spherical particles with cubic anisotropy, *H*_c_ obeys the relation of *H*_c_ = 0.64 *K*/*M*_s_ [15], where *K* is the anisotropy constant, so the *K* value can be calculated for all samples. The highest *K* for CoFe_2_O_4_ NPs at 10 K reaches ~10^7^ erg/cm^3^, much larger than 1.8–3.0 × 10^6^ erg/cm^3^ for the bulk CoFe_2_O_4_; this is consistent with the previous reports that in thin films and nanoparticles, surface anisotropy constant is found higher by many orders of magnitude than that of the bulk [15, 16]. In narue the enhanced *K* value results from the interaction between the surface spins of different particles [7], and between the spins from the surface and core in a single NP [6, 13]. Large *K* value induced by the surface spins leads to the increase of *H*_c_ due to *H*_c_ ∝ *K*. It should be mentioned that the fitting according to Kneller’s law and the calculation of *K* from *H*_c_ = 0.64 *K*/*M*_s_ are based on an assumption that nanoparticles do not interact with each other. The real situation is that the dipolar interaction widely exists in the system of magnetic nanoparticles; however, the dipolar interaction is weak, compared with the anisotropy. Therefore, such the assumption has usually been considered to be reasonable in many previous reports [6, 15, 35].

**Fig. 6:**
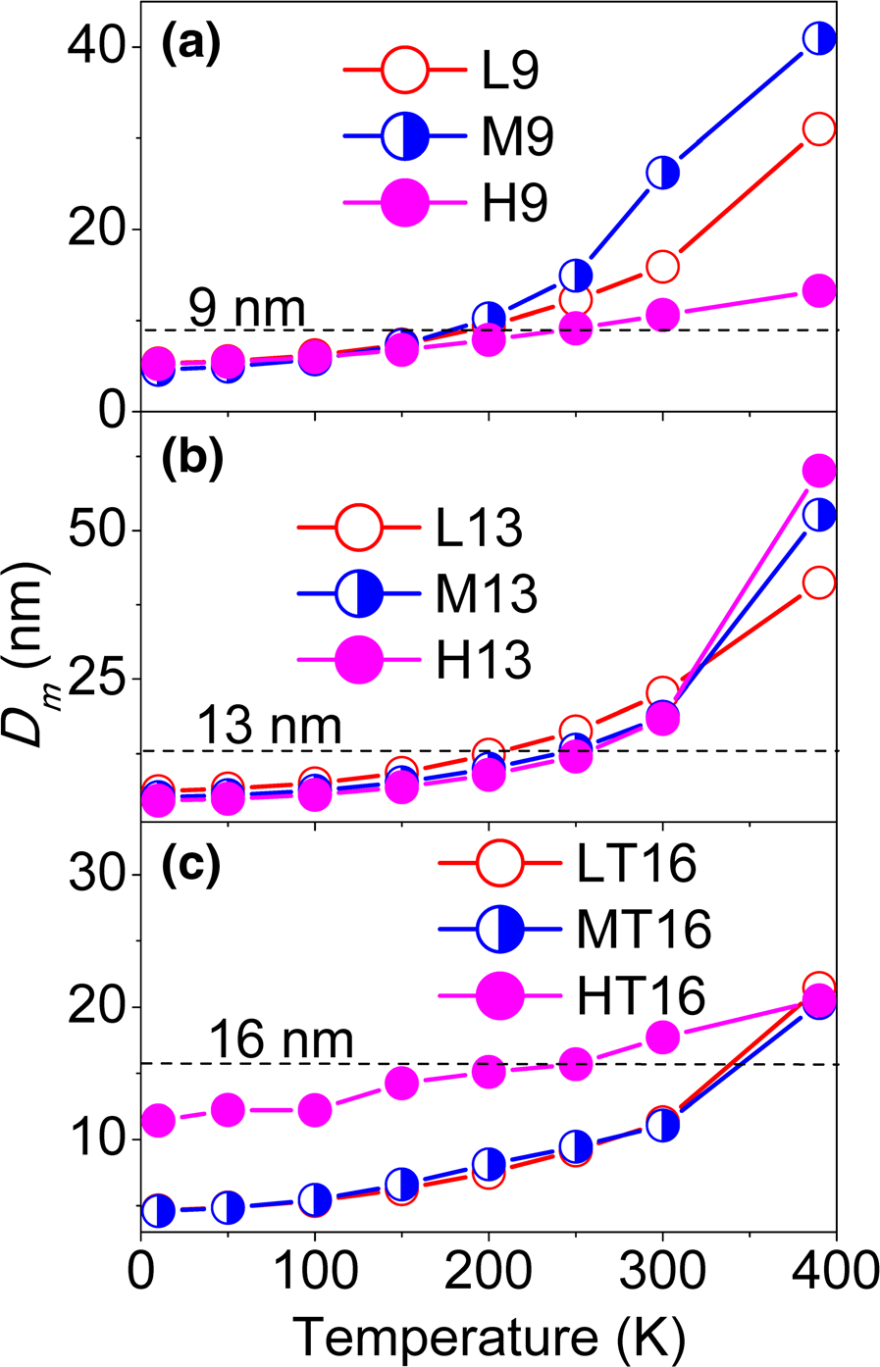
Magnetic grain size *D*_m_ at different temperatures of the diluted 9-nm (**a**) and 13-nm (**b**) NPs, diluted and reduced 16-nm (**c**) NPs

Given *T*_B_ and *K*, the magnetic grain sizes (*V*_m_) of the nanoparticles can be estimated according to Stoner-Wohlfarth expression [36]: 25*k*_B_ *T*_B_ = *KV*_m_, where *k*_B_ is the Boltzmann constant. Subsequently, the diameter *D*_m_ of a magnetic grain for all samples can be obtained, as shown in Fig. 6.

The *D*_m_ values of the 9- and 13-nm NPs are smaller than *D*_TEM_ below about 250 K, because of the canted surface spin layer surrounding magnetic particles [37]. Above this temperature, *D*_m_ becomes larger than *D*_TEM_ because the surface spins become able to thermally fluctuate, so they can be polarized by the core moments [38]. Subsequently, the collective behavior of several particle moments promoted by the IPDI leads to an increase of *D*_m_ [28]. In the case of the diluted and reduced 16-nm NPs, *D*_m_ of the HT16 sample is much larger than that for the LT16 and MT16 samples as a result of the increase of strong magnetic CoFe_2_.

**Fig. 7:**
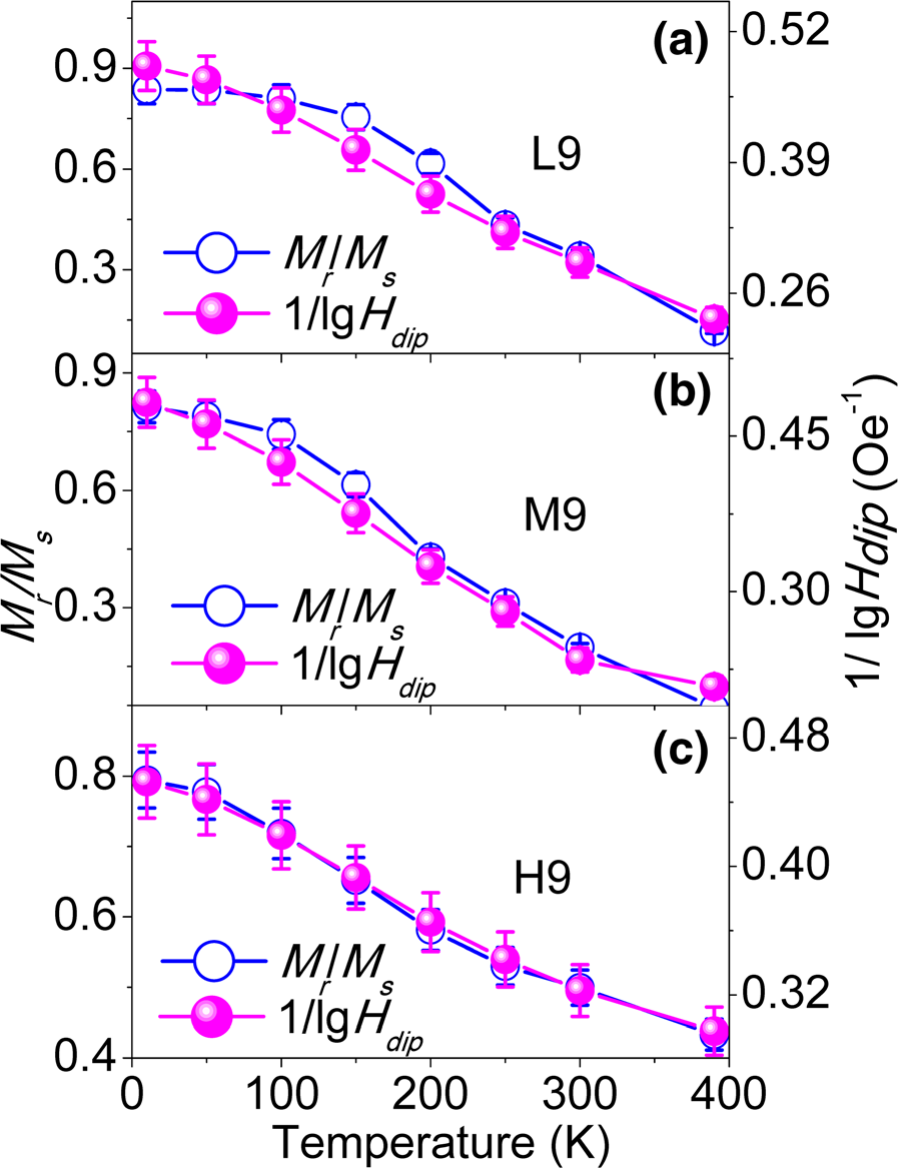
The plot of the 1/lg*H*_dip_ values and *M*_r_/*M*_s_ ratios with added error bars (5 %) against temperature for diluted 9-nm NPs: L9 (**a**), M9 (**b**), and H9 (**c**)

**Fig. 8:**
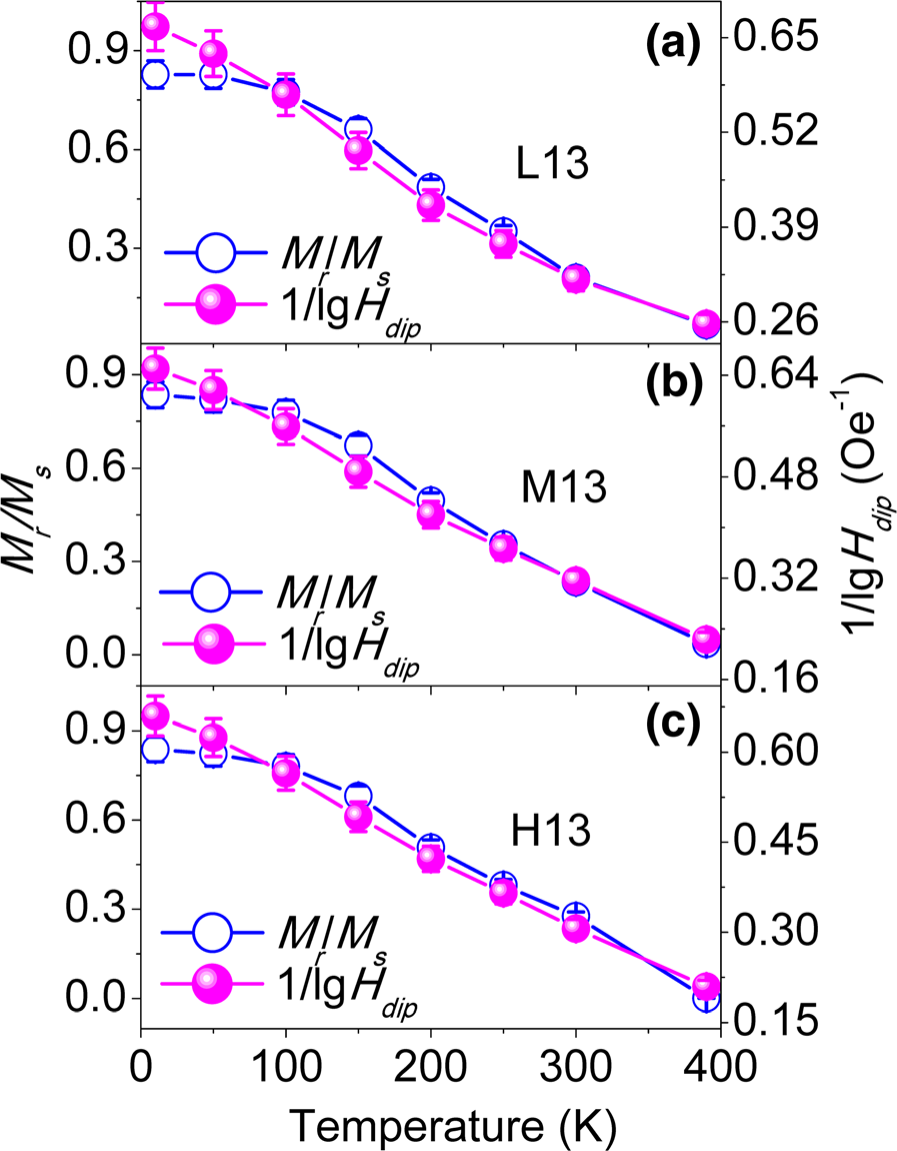
The plot of the 1/lg*H*_dip_ values and *M*_r_/*M*_s_ ratios with added error bars (5 %) against temperature for diluted 13-nm NPs: L13 (**a**), M13 (**b**), and H13 (**c**)

**Fig. 9:**
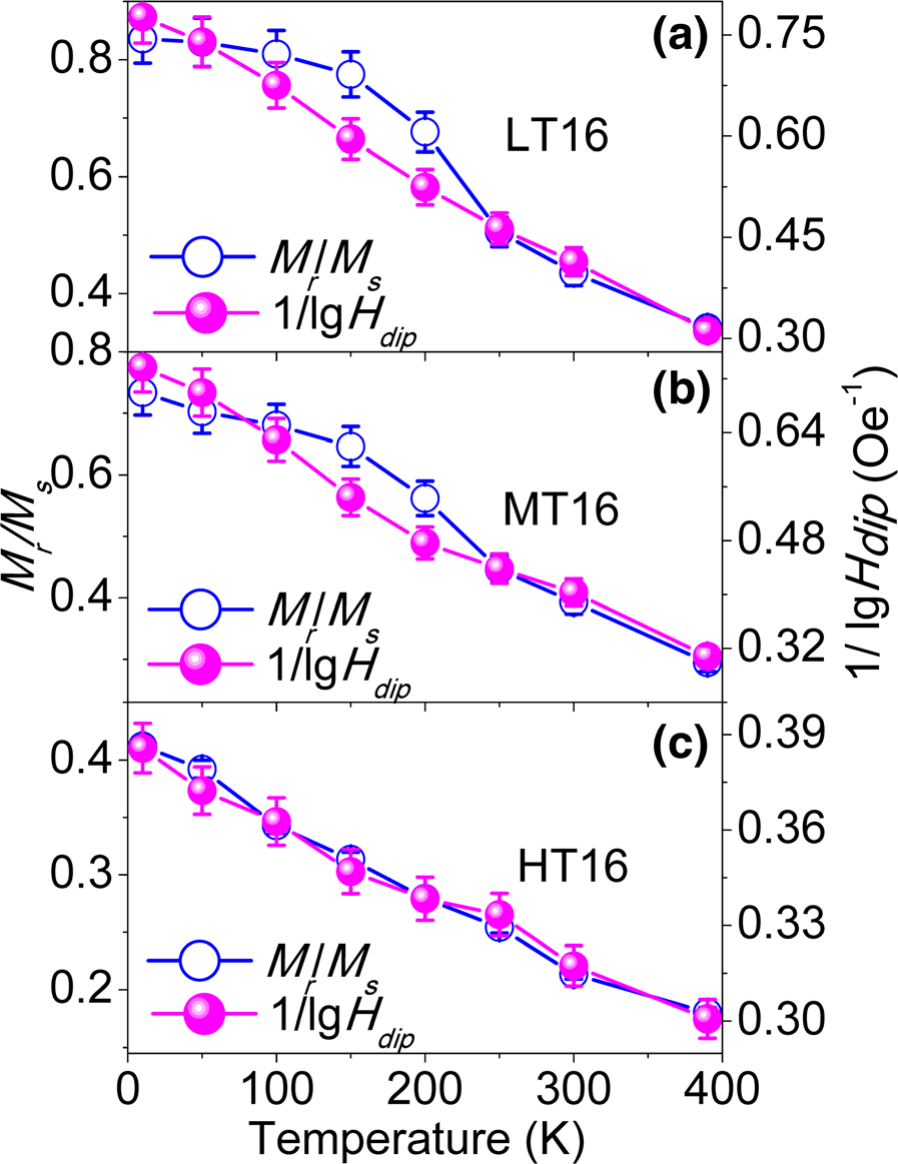
The plot of the 1/lg*H*_dip_ values and *M*_r_/*M*_s_ ratios with added error bars (5 %) against temperature for diluted 16-nm NPs: LT16 (**a**), MT16 (**b**), and HT16 (**c**)

Based on the obtained *V*_m_ values, the strength of IPDI can be estimated by calculating *H*_dip_ according to *H*_dip_ = 2 *μ*/*d* ^3^, where *μ* is the particle moment (*μ* = *M*_s_ × *V*_m_; *M*_s_ is saturation magnetization and *V*_m_ is magnetic grain volume). The inverse of the logarithm of *H*_dip_, i.e., 1/lg*H*_dip_ values and *M*_r_/*M*_s_ ratios with added error bars (5 %) are plotted against temperature (*T*) in Figs. 7, 8, and 9 for 9-, 13-, and 16-nm NPs, respectively.

Next, we will discuss the correlation between *M*_r_/*M*_s_ and *H*_dip_. As seen in Figs. 7 and 8, the correlation between *M*_r_/*M*_s_ and 1/lg*H*_dip_ roughly follows *M*_r_/*M*_s_ ∝ 1/lg*H*_dip_ within a reasonable error range for 9- and 13-nm NPs, even though the NPs have different size and concentration. It can be noticed from Figs. 7 and 8 that the slope of the *M*_r_/*M*_s_ curve is smaller than that for the 1/lg*H*_dip_ curve at low temperatures, resulting in the deviation between *M*_r_/*M*_s_ and 1/lg*H*_dip_, which may result from the several competing effects, including the surface effects, finite size effects, and interparticle interactions, that are sometimes difficult to isolate in these nanoparticles, as suggested by Maaz [6]. As discussed in Fig. 6, the surface spins exist around the NP at low temperatures. Therefore, it is reasonable to suggest that the smaller slope of *M*_r_/*M*_s_ may originate from the interaction between spins of surface and core and between surface spins of the neighboring particles.

**Fig. 10:**
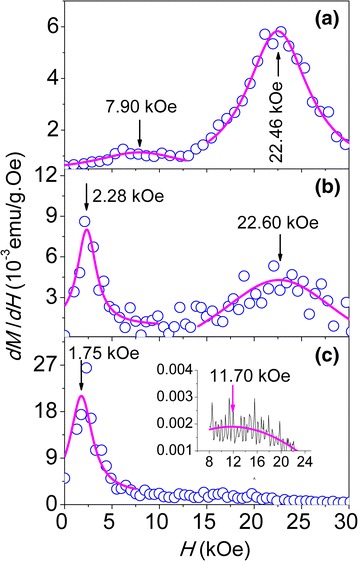
The field derivative *dM*/*dH* of the virgin curves at 10 K for LT16 (**a**), MT16 (**b**), and HT16 (**c**) samples. The *inset* in (**c**) shows local *dM*/*dH* data around 11.70 kOe of the HT16 sample. The *pink solid line* is the curve of Lorentz fit in order to find the peak position

In the case of the diluted and reduced 16-nm NPs which contain CoFe_2_O_4_ and CoFe_2_, as seen in Fig. 9, the obvious deviation between *M*_r_/*M*_s_ and 1/lg*H*_dip_ for the slightly reduced LT16 and MT16, while *M*_r_/*M*_s_ matches well with 1/lg*H*_dip_ for the heavily reduced HT16, which can be assigned to the interaction in CoFe_2_O_4_/CoFe_2_ NPs. The interactions in CoFe_2_O_4_/CoFe_2_ NPs occur at the interface (intraparticle) of CoFe_2_O_4_ and CoFe_2_ and from the contact between NPs (interparticle). CoFe_2_O_4_ has a large magnetic anisotropy, so that it can exert a pinning action on the CoFe_2_ phase, similar to the pinning effect of Fe oxide on Fe in Fe/Fe oxide NPs [38], which is the possible reason for the deviation between *M*_r_/*M*_s_ and 1/lg*H*_dip_. This suggestion may be supported by the results of the irreversible magnetization reversal field (*H*_irr_) [27], defined as the magnetic field where the derivative (*dM*/*dH*) of the virgin curve has a peak. Figure 10 shows the field derivative *dM*/*dH* of the virgin curves for LT16 (a), MT16 (b), and HT16 (c) samples at 10 K. Two peaks locate at *H*_irr_ = 7.90 and 22.46 kOe for the LT16 sample, at *H*_irr_ = 2.28 and 22.60 kOe for the MT16 sample, and at *H*_irr_ = 1.75 and 11.70 kOe for the HT16 sample. The lower field corresponds to *H*_irr_ of CoFe_2_, because pure CoFe_2_ is a typical soft magnet and its *H*_irr_ is about 0.9 kOe at 10 K (not shown here), while the higher field corresponds to *H*_irr_ of hard CoFe_2_O_4_. The LT16 sample contains more CoFe_2_O_4_ and less CoFe_2_, and CoFe_2_O_4_ exerts the pinning effect on the moment of CoFe_2_, leading to the highest *H*_irr_ of CoFe_2_ among three samples. With increasing the reduction temperature, the sample contains more CoFe_2_, and therefore the moment of CoFe_2_ cannot be fully pinned by CoFe_2_O_4_, leading to the lower *H*_irr_ of CoFe_2_ for the samples MT16 and HT16. Possibly, more CoFe_2_ in HT16 polarizes the moments of CoFe_2_O_4_, resulting in the smaller *H*_irr_ of CoFe_2_O_4_ than that of LT16 [38]. These interactions in CoFe_2_O_4_/CoFe_2_ NPs affect the moment reversal and consequently affect the *M*_r_/*M*_s_ ratio.

However, compared with LT16 and MT16, the heavily reduced HT16 has the higher CoFe_2_ content as a result of reduction at a higher temperature 500 °C. Therefore, HT16 has larger particle moment, and hence stronger interparticle dipolar interaction which overcomes other effects such as surface spins and interaction between CoFe_2_O_4_ and CoFe_2_, consequently making the correlation between *M*_r_/*M*_s_ and 1/lg*H*_dip_ obey *M*_r_/*M*_s_ ∝ 1/lg*H*_dip_.

## Conclusions

Well-dispersed uniform CoFe_2_O_4_ NPs with sizes of 9-, 13-, and 16-nm were synthesized. Some 9- and 13-nm NPs were diluted in a SiO_2_ matrix to change their concentration that is inversely proportional to interparticle distance, and some diluted 16-nm NPs were reduced by H_2_ at 300, 400, and 500 °C to change the moment and anisotropy of the NPs. These samples were used as model systems to reveal the intrinsic correlation between *M*_r_/*M*_s_ and IPDI, the strength of which was estimated by *H*_dip_.

For the diluted 9- and 13-nm NPs that were not reduced, the correlation between *M*_r_/*M*_s_ and *H*_dip_ follows *M*_r_/*M*_s_ ∝ 1/lg *H*_dip_, regardless of the particle size and distance. Slight deviation from *M*_r_/*M*_s_ ∝ 1/lg *H*_dip_, occurring at low temperatures, can be attributed to the effects of surface spins. In the case of the diluted and reduced 16-nm NPs, the relation between *M*_r_/*M*_s_ and *H*_dip_ deviates *M*_r_/*M*_s_ ∝ 1/lg*H*_dip_ for the slightly reduced NPs at 300 and 400 °C because of the pinning effect of CoFe_2_O_4_ on CoFe_2_. However, the heavily reduced NPs at 500 °C follows *M*_r_/*M*_s_ ∝ 1/lg *H*_dip_ because the strong interparticle dipolar interaction is a dominant factor to affect *M*_r_/*M*_s_.

## Abbreviations

(HR) TEM: (High-resolution) transmission electron microscopy
IPDI: Interparticle dipolar interaction
PDF: Standard powder diffraction file
SSG: Super-spin glass
XRD: X-ray diffraction
